# Real-Time and Selective Detection of *Pseudomonas aeruginosa* in Beef Samples Using a g-C_3_N_4_-Doped Multimetallic Perovskite-Based Electrochemical Aptasensor

**DOI:** 10.3390/bios15100634

**Published:** 2025-09-23

**Authors:** Sarah S. Albalawi, Naeem Akhtar, Waleed A. El-Said

**Affiliations:** 1 Department of Chemistry, College of Science, University of Jeddah, P.O. Box 80327, Jeddah 21589, Saudi Arabia; 2 Institute of Chemical Sciences, Bahauddin Zakariya University (BZU), Multan 60800, Pakistan

**Keywords:** foodborne diseases, biosensor, bacteria, *Pseudomonas aeruginosa*, graphitic carbon nitride, metal oxides, aptasensor

## Abstract

The alarming rise in foodborne illnesses, particularly those associated with microbial contamination in meat products, presents a serious challenge to global food safety. Among these microbial threats, *Pseudomonas aeruginosa* (*P. aeruginosa*) poses a critical threat due to its biofilm-forming capability and prevalence in contaminated beef, highlighting its effective real-time detection. Herein, we report the fabrication of a novel electrochemical aptasensor based on multimetal perovskite (FeCoCuNiO) doped with urea-derived graphitic carbon nitride (g-C_3_N_4_), synthesized via a sol–gel combustion method. The FeCoCuNiO-g-C_3_N_4_ nanocomposite was then coated onto a graphitic pencil electrode and functionalized with a DNA-based aptamer specific towards *P. aeruginosa*. The resulting aptasensor exhibited a low detection limit of 3.03 CFU mL^−1^ with high selectivity and sensitivity, and was successfully applied to real-time detection of *P. aeruginosa* in food samples. To the best of our knowledge, this work presents the first FeCoCuNiO-g-C_3_N_4_-based aptasensor for bacterial detection, offering a promising platform for food safety assurance and public health protection.

## 1. Introduction

Foodborne diseases continually impact consumers, health authorities, and the food industry. Among various food products, meat, especially beef, is highly perishable, with over 20% of global meat production wasted due to microbial spoilage [1]. Fresh meat and meat products are common sites for foodborne pathogens [2]. Contamination of beef can occur through multiple routes, including cross-contamination during slaughter, unsanitary processing environments, and inadequate cold-chain practices [3]. Bacteria, the second leading cause of foodborne illnesses, pose significant threats, with approximately 3.6 million cases reported annually [4]. Of these, 1.5 million are caused by diarrheagenic Escherichia coli, Salmonella, and *Pseudomonas aeruginosa* (*P. aeruginosa*). *P. aeruginosa* is especially concerning because it causes gastrointestinal infections, diarrhea, abdominal pain, and nausea [5]. This highly adaptable Gram-negative bacterium threatens public health and food safety due to its inherent antibiotic resistance and ability to form biofilms rapidly [6]. This alarming adaptability highlights the urgent need for rapid, sensitive, and specific detection of *P. aeruginosa* at low concentrations for real-time analysis [7].

In recent years, several methods have been adopted for the detection of *P. aeruginosa*, including magnetic relaxation switch aptasensor [8] surface-enhanced Raman spectroscopy [9], colorimetric assays [10,11], and polymerase chain reaction techniques [12]. Although these approaches are highly sensitive and accurate, their use in bacterial detection is often limited due to long processing times, the need for specialized instruments, and complex operational procedures [13]. This highlights the need for faster, cost-effective, and user-friendly methods for the reliable diagnosis of *P. aeruginosa*. Electrochemical sensors have recently gained considerable attention due to their advantages, including low cost, rapid detection, ease of use, and high sensitivity [14,15]. Among these, electrochemical aptasensors stand out for their ability to provide rapid, selective, and sensitive detection of pathogens [16,17]. A key factor in improving the performance of aptasensors lies in the choice of electrode materials. An ideal electrode material should promote fast electron transfer, ensure strong and stable aptamer immobilization, and support low detection limits and high sensitivity.

In the past few decades, noble metals such as Pt [18], Pd [19], Ag [20], and Au [21]-based nanocomposites have been used for pathogen detection due to their high sensitivity and excellent stability. However, their high cost, limited abundance, and poor scalability have significantly hindered their widespread commercial applications [22]. In this regard, a diverse range of transition metal-based materials, including oxides [23], phosphides [24], nitrides [25], sulfides [26], and perovskites, have emerged as promising electrochemical sensors [27]. Among these, perovskite-type oxides (ABO_3_) have attracted considerable attention owing to their structural versatility, tunable electronic properties, and catalytic adaptability [28]. Despite these advantages, perovskites still face inherent limitations, such as relatively poor electrical conductivity and restricted exposure of active sites due to agglomeration or limited porosity [29]. To address these challenges, various conductive materials such as purines, polyaniline [30], poly (3,4-ethylene dioxythiophene) [31], urea [32], and polypyrrole have been reported [33]. Among these conductive materials, urea-derived graphitic carbon nitride (g-C_3_N_4_) has garnered significant attention due to its high electrical and thermal conductivity, good mechanical and chemical stability, high surface area, and favorable carrier transport [34,35]. Therefore, doping g-C_3_N_4_ with transition metal-based perovskite is expected to enhance its electrochemical performance and stability.

**Scheme 1:**
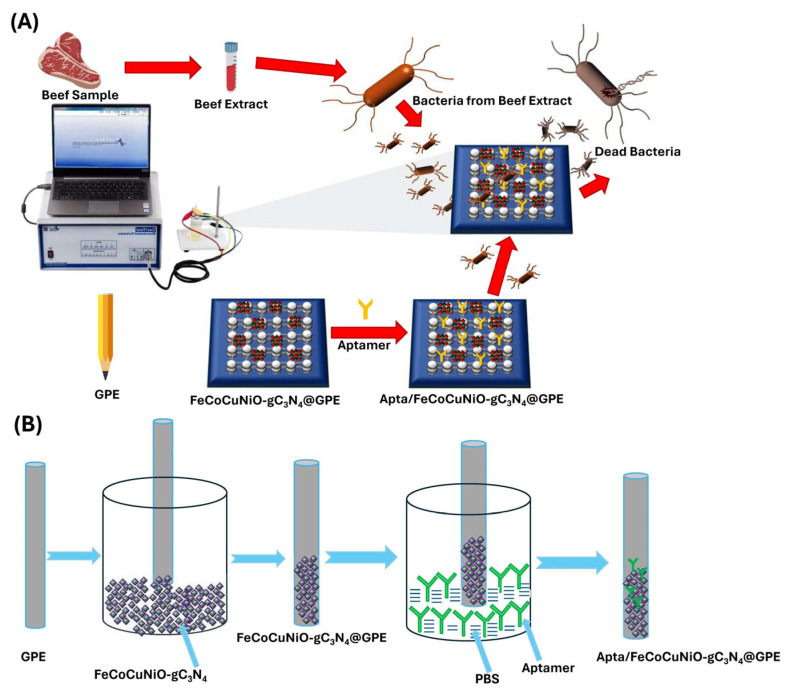
(**A**) Development of *P. aeruginosa* electrochemical biosensor and its utilization for sensing in beef samples, and (**B**) fabrication of the aptasensor.

Thus, herein, we synthesized FeCoCuNiO via a sol–gel method and subsequently doped with g-C_3_N_4_ to design the electrochemical sensor (FeCoCuNiO-g-C_3_N_4_) for the sensitive detection of *P. aeruginosa*. To further proceed with the electrochemical applications, the as-synthesized FeCoCuNiO-g-C_3_N_4_ material was coated on a graphitic pencil electrode (GPE). To improve the selectivity of the designed sensor, the FeCoCuNiO-g-C_3_N_4_/GPE electrode was functionalized with DNA-based aptamers specific to *P. aeruginosa.* g-C_3_N_4_ not only enhanced the electrochemical conductivity of the sensor platform but also facilitated efficient and stable immobilization of the aptamer. Scheme 1 shows the development of g-C_3_N_4_-doped FeCoCuNiO-modified graphitic pencil electrode (FeCoCuNiO-g-C_3_N_4_/GPE)-based aptasensor and its use for *P. aeruginosa* detection in beef samples. The resulting aptasensor exhibited excellent electrocatalytic activity and achieved a low limit of detection (LOD) of 3.03 CFU mL^−1^. Furthermore, the FeCoCuNiO-g-C_3_N_4_/GPE-based electrochemical sensor was successfully employed for the real-time detection of *P. aeruginosa* in food samples. As far as we know, this is the first report describing a FeCoCuNiO-g-C_3_N_4_-based aptasensor for *P. aeruginosa* detection, highlighting its potential as a powerful tool for food safety measurements and environmental monitoring applications.

## 2. Materials and Methods

### 2.1. Materials

Copper(II) acetate monohydrate (Cu(CH_3_COO)_2_·H_2_O), ferric nitrate nonahydrate (Fe(NO_3_)_3_·9H_2_O), ethylenediaminetetraacetic acid (EDTA, C_10_H_16_N_2_O_8_), aqueous ammonium hydroxide solution (NH_3_, 28%), nickel(II) acetate tetrahydrate (Ni(CH_3_COO)_2_·4H_2_O), phosphate-buffered saline (PBS), and citric acid were all obtained from Sinopharm Chemical Reagent Co., Ltd (Shanghai, China). All the reagents were of analytical grade and used as received without further purification. The graphitic pencil electrode (GPE) with a 0.5 mm dimension and 0.0039 cm^2^ geometric surface area was purchased from the local market, Lahore, while the reference and counter electrodes were purchased from Gamry Instruments(Pennsylvania, USA). All bacterial strains, including *Salmonella enterica*, *Klebsiella pneumoniae* (*K. pneumoniae*), *Escherichia coli* (*E. coli*), and *P. aeruginosa*, were obtained from the Department of Pharmacology, Nishtar Medical University, Multan, Pakistan. *P. aeruginosa* aptamer sequence 5′-CCC CCG TTG CTT TCG CTT TTC CTT TCG CTT TTG TTC GTT TCG TCC CTG CTT CCT TTC TTG-3′ with Bio-RP purification was purchased from Bioneer Company, (Daejeon) South Korea, in the form of a lyophilized powder.

### 2.2. Synthesis of g-C_3_N_4_

Graphitic carbon nitride (g-C_3_N_4_) was synthesized via thermal polymerization of urea, following a previously reported method [36]. Briefly, 5.0 g of urea powder was finely ground to ensure uniform particle size. The obtained powder was transferred to a covered alumina crucible and subjected to calcination in a muffle furnace at 550 °C for 2 h under ambient conditions. After natural cooling to room temperature, a yellow-colored powder was collected, indicating the successful formation of g-C_3_N_4_. The product was stored in a desiccator and used for further processing.

### 2.3. Synthesis of Quaternary Metallic (FeCoCuNiO) Perovskite

A sol–gel combustion approach was used to synthesize the quaternary transition metal-based perovskite (FeCoCuNiO) [37]. Stoichiometric amounts of CoCl_2_·6H_2_O, Ni(CH_3_COO)_2_·4H_2_O, Cu(CH_3_COO)_2_·H_2_O, and Fe(NO_3_)_3_·9H_2_O were dissolved in deionized water under continuous stirring. Consequently, citric acid and EDTA were then added into the solution in a molar ratio of 2:1 (citric acid–EDTA) as complexing and chelating agents, respectively. Then the aqueous ammonium hydroxide (28%) was added to maintain the pH of the solution, and the reaction mixture was continuously stirred at 90 °C until a viscous gel formed. The obtained gel was thermally treated at 250 °C to eliminate residual moisture. Finally, the dried product was calcined at 800 °C for 10 h in air to obtain the desired crystalline FeCoCuNiO perovskite nanocomposite.

### 2.4. Synthesis of g-C_3_N_4_-Doped FeCoCuNiO Nanocomposite

The g-C_3_N_4_-doped quaternary metal oxide nanocomposite (FeCoCuNiO-g-C_3_N_4_) was synthesized via a thermal doping approach [38]. Briefly, 0.7 g of the previously synthesized FeCoCuNiO powder was thoroughly mixed with 5.0 g of urea using a mortar and pestle to obtain a homogeneous mixture. Pyrolysis was carried out by heating the mixture in a covered crucible at 550 °C for 2 h in a muffle furnace under an ambient air atmosphere. During this process, urea was then decomposed to form g-C_3_N_4_, which simultaneously integrated with the FeCoCuNiO phase. The obtained yellow-brownish powder was collected and stored for further application.

### 2.5. Fabrication of g-C_3_N_4_-Doped FeCoCuNiO Electrode

Prior to modification, the graphitic pencil electrode (GPE) was rinsed with deionized water to ensure a clean and smooth surface. A uniform suspension of the FeCoCuNiO-g-C_3_N_4_ was prepared, followed by ultrasonication for 20–30 min to achieve a stable and homogeneous mixture. Then, the GPE was suspended in an as-prepared suspension to prepare the FeCoCuNiO-g-C_3_N_4_/GPE [39]. The modified electrode (FeCoCuNiO-g-C_3_N_4_/GPE) was then rinsed with deionized water to remove unbound material and then dried before use.

### 2.6. Fabrication of Aptasensor

Firstly, a stock solution of aptamer (20 µL of 0.5 µM) was prepared in Tris buffer (10 mM Tris, 0.1 mM EDTA, 1 mM NaCl_2_, at pH 8.0) and then stored at 4 °C overnight to form the self-assembled monolayer. Furthermore, a series of aptamer dilutions (0.1 to 0.5 nM) was prepared in 0.1 M PBS to facilitate subsequent experimental evaluations. To immobilize the aptamer on the surface of the FeCoCuNiO-g-C_3_N_4_/GPE electrode, the fabricated FeCoCuNiO-g-C_3_N_4_/GPE electrode was incubated in aptamer solutions to enable surface functionalization and then dried overnight to obtain the aptamer immobilized FeCoCuNiO-g-C_3_N_4_/GPE (aptasensor). Finally, the fabricated aptasensor was exposed to varying concentrations of *P. aeruginosa* for the electrochemical detection method, including CV, for EIS measurements in 0.1 M PBS electrolyte containing 1 mM of [Fe(CN)_6_]^4−/3−^.

### 2.7. Bacterial Strains and Cultivation

The cultivation of *P. aeruginosa* was carried out in Luria Broth (LB) at 37 °C for 18 h under aerobic conditions, following a previously reported protocol [15]. The bacterial concentration of the resulting culture was estimated based on the McFarland Turbidity Standard [40], allowing for the approximation of colony-forming units per milliliter (CFU mL^−1^). Subsequently, serial dilutions 10^1^, 10^2^, 10^3^, 10^4^, 10^5^, 10^6^, and 10^7^ CFU mL^−1^ from the stock culture were prepared using 0.1 mol L^−1^ PBS (pH 7.4). All procedures were carried out under aseptic conditions to prevent contamination and ensure the reliability of experimental results.

### 2.8. Morphological, Compositional, and Electrochemical Characterizations

Structural and morphological characterizations of the synthesized nanocomposite were carried out by X-ray diffraction (XRD), Fourier transform infrared spectroscopy (FTIR), and scanning electron microscopy (SEM). Furthermore, electrochemical performance was evaluated by cyclic voltammetry (CV) and electrochemical impedance spectroscopy (EIS) using a three-electrode system potentiostat. Moreover, the instruments have been provided in the Supplementary Materials Section S1.

## 3. Results and Discussion

### 3.1. Composition and Structural Insights of Synthesized Materials

**Figure 1:**
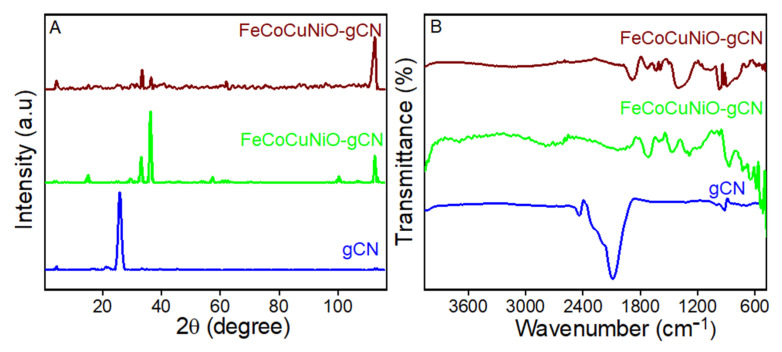
The (**A**) XRD of g-C_3_N_4_, FeCoCuNiO-g- C_3_N_4_, and FeCoCuNiO-g-C_3_N_4_, and (**B**) FTIR spectra of C_3_N_4_, FeCoCuNiO-g-C_3_N_4_, and FeCoCuNiO-g- C_3_N_4_.

The structural and chemical properties of the synthesized materials were characterized using X-ray diffraction (XRD) and Fourier-transform infrared spectroscopy (FTIR), as illustrated in Figure 1. The XRD spectrum of g-C_3_N_4_ (Figure 1A, blue line) shows a sharp peak near 27.4° (2θ), corresponding to the (002) reflection, characteristic of interlayer stacking in g-C_3_N_4_. The peak confirms the presence of graphitic-layered structures formed by π–π stacking of conjugated planes. Multiple peaks between 20° and 80° (2θ) represent the crystalline nature of mixed-metal oxides (Figure 1A, green line). The absence of a sharp peak at 27.4° confirms no g-C_3_N_4_ contribution in this spectrum. These peaks likely correspond to NiO, CuO, Co_3_O_4_, and Fe_2_O_3_ phases, often observed in spinel or mixed oxide structures. Peaks corresponding to g-C_3_N_4_ (002) and metal oxides show the diffraction planes of (111), (200), (320), and (510), confirming the successful integration of g-C_3_N_4_ with FeCoCuNiO. Broadened peaks suggest reduced crystallite size or partial amorphization due to composite formation (Figure 1A, maroon line).

FTIR analysis further supports these findings. The spectrum of g-C_3_N_4_ shows a distinct absorption band at ~810 cm^−1^ corresponding to the breathing mode of s-triazine units, along with stretching vibrations of C–N and C=N bonds in the 1200–1650 cm^−1^ range (Figure 1B, blue line). A broad N–H stretching band near 3100–3300 cm^−1^ was also observed, arising from residual –NH or –NH_2_ groups. For FeCoCuNiO, absorption bands associated with -OH stretching (~3400 cm^−1^), symmetric/asymmetric COO^−^ stretching (~1400–1600 cm^−1^), and strong M-O vibrations below 700 cm^−1^ were clear, confirming the presence of surface hydroxyl groups, residual carboxylates [41], and metal–oxygen bonds, respectively (Figure 1B, green line). In the composite FeCoCuNiO-g-C_3_N_4_ spectrum, both g-C_3_N_4_ and metal oxide features were observed, with enhanced M-O stretching signals and slight shifts in peak positions, indicating strong interfacial interactions between the g-C_3_N_4_ and metal oxide species (Figure 1B, maroon line).

**Figure 2:**
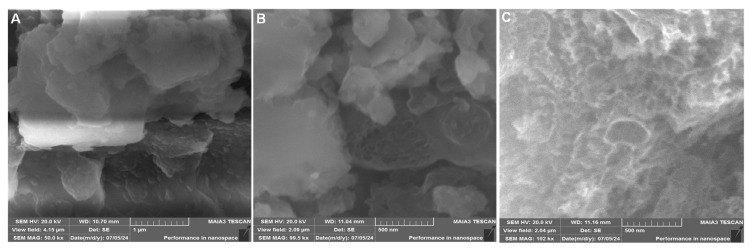
SEM images of (**A**) FeCoCuNiO and (**B**) FeCoCuNiO-g-CN nanocomposite. (**C**) High-resolution image of FeCoCuNiO-g-CN nanocomposite.

The surface morphology of the synthesized materials was examined using scanning electron microscopy (SEM), as shown in Figure 2. The FeCoCuNiO nanoparticles display a significantly different morphology of irregular-shaped, aggregated crystalline particles with relatively rough and dense surfaces, with a diameter of 210–260 nm (Figure 2A). The observed dense agglomeration suggests strong interparticle interactions, as previously supported by XRD data. The FeCoCuNiO-g-C_3_N_4_ nanocomposite reveals a successful modification of g-C_3_N_4_ over the surface of metal oxide nanoparticles, forming a heterostructured architecture (Figure 2B). Notably, the hybrid composite exhibits an increased surface roughness and well-defined interfaces between the C_3_N_4_ support and the FeCoCuNiO, with particle size ranging from 180 to 220 nm. This uniform dispersion of C_3_N_4_ over the layered oxide nanoparticles not only confirms strong physical interaction but also suggests improved electroactive sites. The improved resolution in Figure 2C provides clear evidence of the successful surface modification and uniform dispersion of g-C_3_N_4_ over the FeCoCuNiO matrix (Figure 2C).

### 3.2. Electrochemical Behavior of Designed Electrode

**Figure 3:**
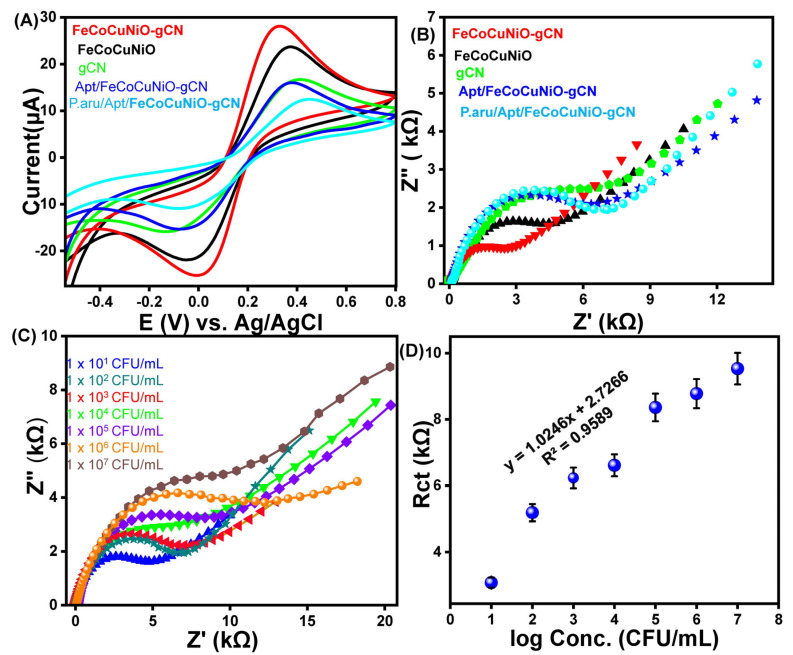
The (**A**) CV and (**B**) EIS spectra of fabricated electrodes towards *P. aeruginosa* detection in 0.1 M PBS electrolyte containing 1 mM of [Fe(CN)_6_]^4−/3−^. (**C**) EIS spectra of the designed aptasensor at different concentrations of *P. aeruginosa* (1 × 10^1^–1 × 10^7^ CFU/mL) and (**D**) the corresponding calibration curve of Rct versus logarithm of concentration of *P. aeruginosa*.

Electrochemical properties of fabricated g-C_3_N_4_, FeCoCuNiO, FeCoCuNiO-g-C_3_N_4_, Apt/FeCoCuNiO, and *P. aeruginosa*/Apt/FeCoCuNiO electrodes were investigated to study the electron transfer resistance by cyclic voltammetry (CV) and electrochemical impedance spectroscopy (EIS) using 0.1 M PBS electrolyte containing 1 mM of [Fe(CN)_6_]^4−/3−^ (Figure 3). The CV curves show that FeCoCuNiO-g-C_3_N_4_ exhibits a maximum current response (28.3 μA) compared to FeCoCuNiO (23.8 μA) and g-C_3_N_4_ (16.8 μA), indicating the superior electron transfer efficiency of FeCoCuNiO-g-C_3_N_4_ due to the synergistic effect between the multimetallic oxide and g-C_3_N_4_. In contrast, the bare FeCoCuNiO electrode showed moderate current intensity, reflecting its inherent electroactivity. The g-C_3_N_4_-modified electrode displayed relatively low current, likely due to its limited electrical conductivity. Similarly, the distinct differences were observed in the peak-to-peak separations (ΔEp) among the modified electrodes FeCoCuNiO-g-C_3_N_4_ (0.31 V), FeCoCuNiO (0.33 V), and g-C_3_N_4_ (0.35 V), reflecting their respective charge transfer kinetics and interfacial properties. Upon functionalization of the FeCoCuNiO-g-C_3_N_4_ electrode with aptamer Apt/FeCoCuNiO-g-C_3_N_4_, a noticeable decrease in the redox peak current (16.2 μA) and an increase in ΔEp (0.3 V) were observed, which is attributed to the introduction of an insulating biomolecular layer that impedes electron transfer between the electrode surface and the redox probe. Following the specific binding of *P. aeruginosa* to the aptamer-functionalized surface, a further suppression in current (12.6 μA) and an increase in ΔEp (0.37 V) response were recorded. This additional decline confirms the successful recognition event and suggests the formation of a compact layer that acts as a diffusion barrier, thereby restricting the accessibility of electroactive species to the electrode interface (Figure 3A). Furthermore, EIS measurements were carried out to study the charge transfer resistance (Rct) of the fabricated electrodes. The Nyquist plots of the modified electrodes have demonstrated that FeCoCuNiO-g-C_3_N_4_ exhibits the smallest semicircle, indicating the lowest Rct (2.6 kΩ) value as compared to FeCoCuNiO Rct (4.53 kΩ) and g-C_3_N_4_Rct (6.05 kΩ). The lowest Rct value of FeCoCuNiO-g-C_3_N_4_ confirms the enhanced conductivity, while FeCoCuNiO and g-C_3_N_4_ together facilitate the faster electron transfer at the electrode interface. In contrast, the Apt/FeCoCuNiO-g-C_3_N_4_ electrode shows a larger semicircle with high resistivity (4.83 kΩ), due to the presence of an aptamer layer that introduces a non-conductive barrier, hindering electron exchange between the electrode and redox species. Furthermore, the *P. aeruginosa*/Apt/FeCoCuNiO-g-C_3_N_4_ electrode demonstrates the largest semicircle, reflecting the highest Rct (6.05 kΩ), which confirms successful binding of the *P. aeruginosa* target. This biological interaction likely forms a thick insulating layer, acting as a diffusion and electron transfer barrier, thereby significantly impeding charge transport (Figure 3B).

### 3.3. Optimization of Biorecognition Layer

Optimization of the aptamer concentration was carried out to ensure the maximum sensor sensitivity by achieving efficient surface coverage of the electrode without influencing the target–analyte interactions. For this purpose, the EIS was conducted to determine the optimal concentration of aptamer for sensor functionalization, using five concentrations ranging from 1 to 5 nM (Figure S1). The result shows that at a low concentration of aptamer at 1 nM, the fabricated electrode shows the lowest Rct, indicating the most efficient electron transfer and favorable aptamer surface coverage. At concentrations exceeding 1 nM, a progressive increase in Rct was observed, likely due to excessive aptamer adsorption leading to surface saturation. This over-coverage may obstruct the electrode’s active sites, thereby impeding target–analyte interactions, diminishing the overall sensitivity of the fabricated electrode. Consequently, 1 nM was identified as the optimal aptamer concentration and was employed in all subsequent analyses.

### 3.4. Sensing and Selective Performance of the Aptasensor

The analytical performance of the fabricated Apt/FeCoCuNiO-g-C_3_N_4_ aptasensor was evaluated for the quantitative detection of *P. aeruginosa* across a concentration range of 1 × 10^1^ to 1 × 10^7^ CFU mL^−1^ using EIS under optimized conditions, as shown in Figure 3C. Further, the Rct was determined by fitting the EIS data to a Randles equivalent circuit model using the Zview software (Figure S2). The results show that the Rct values increase with increasing concentration of *P. aeruginosa* from 6.2 kΩ (1 × 10^1^ CFU mL^−1^) to 7.9 kΩ (1 × 10^2^ CFU mL^−1^), 8.8 kΩ (1 × 10^3^ CFU mL^−1^), 10.6 kΩ (1 × 10^4^ CFU mL^−1^), 11.4 kΩ (1 × 10^5^ CFU mL^−1^), 14.1 kΩ (1 × 10^6^ CFU mL^−1^), and reach a maximum of 18.3 kΩ (1 × 10^7^ CFU mL^−1^). This behavior is attributed to the effective capture of bacterial cells by the surface-immobilized aptamers, leading to the formation of a non-conductive, sterically hindering biofilm that impedes electron transfer between the redox probe and the electrode surface. To assess the sensitivity and linear range of the sensor, a calibration curve was drawn by correlating the Rct values with the logarithm of *P. aeruginosa* concentrations within the tested range (1.5 × 10^1^–1.5 × 10^7^ CFU mL^−1^), as shown in Figure 3D. The resulting linear regression equation was Rct = 1.0246x + 2.7266, with a correlation coefficient (R^2^) of 0.9589, demonstrating good linearity and a limit of detection (LOD) of 3.03 CFU mL^−1^. These results highlight the excellent sensitivity and linear detection range of the developed aptasensor, underscoring its potential for reliable and ultra-sensitive detection of *P. aeruginosa* in clinical perspectives.

**Figure 4:**
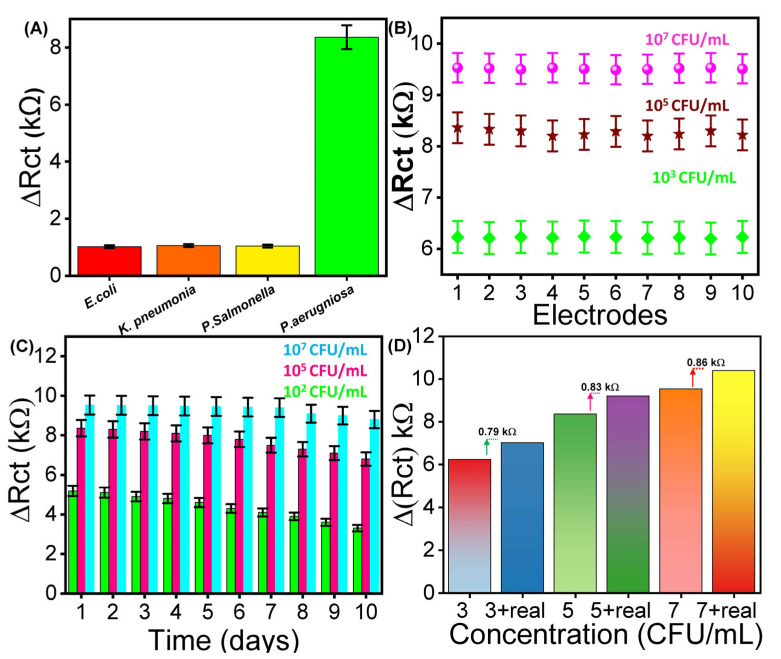
The (**A**) electrochemical detection of bacteria using the fabricated aptasensor in terms of (**A**) selectivity, (**B**) reproducibility, (**C**) reusability, and (**D**) real-time analysis in beef samples.

To evaluate the selectivity of the fabricated aptasensor, it was challenged with different bacterial strains, including Salmonella enterica, K. pneumoniae, E. coli, and *P. aeruginosa*, each at a concentration of 10^5^ CFU mL^−1^ under identical conditions (Figure 4A). The results revealed a negligible Rct response to the non-target strains, whereas a noticeable increase in Rct was observed upon addition to *P. aeruginosa*. This distinct response underscores the high specificity of the aptamer towards *P. aeruginosa*, confirming the excellent selectivity of the developed aptasensor towards the targeted bacterial strain.

### 3.5. Stability Test of the Designed Aptasensor

Stability of the working electrode is a crucial parameter influencing the long-term reliability of electrochemical aptasensors. To assess this, EIS analysis was performed to assess the reproducibility by fabricating ten different electrodes to evaluate the ∆Rct response at a fixed concentration of *P. aeruginosa* (10^3^, 10^5^, and 10^7^ CFU mL^−1^) (Figure 4B). The results show a slight variation in ∆Rct across all ten electrodes at each concentration, resulting in an RSD value of 0.59, 0.684, and 0.72%. Additionally, reusability of the designed aptasensor was evaluated by performing EIS against ten consecutive measurements to evaluate the Rct of the same electrode (Figure 4C). The fabricated electrode was stored at 4 °C and evaluated daily using a fixed concentration of 10^2^, 10^5^, and 10^7^ CFU mL^−1^. The results demonstrate a decrease of 1.11, 1.32, and 1.38% with an RSD of 0.75. The findings clearly demonstrate excellent performance of the developed aptasensor for future applications.

### 3.6. Real Sample Analysis

The practical application of the designed aptasensor for detecting *P. aeruginosa* was investigated using the standard addition method in a real sample (beef) by employing EIS analysis [42]. The beef sample was prepared by following the already reported strategy (please see details in Section S2). Different concentrations of cultured *P. aeruginosa* (10^3^, 10^5^, and 10^7^ CFU mL^−1^) were spiked into 1 mM [Fe(CN)_6_]^4−^/^3−^ containing 0.1 M PBS, and the corresponding ΔRct responses were recorded. Subsequently, the same concentrations were added together with the beef sample, which resulted in slightly higher ΔRct values (0.79, 0.83, and 0.86 kΩ, respectively), as shown in Figure 4D. The results exhibited a noticeable increase in the Rct response, along with real sample recovery rates of 98% to 104% with an RSD ranging from 1.40% to 2.77%, as shown in Table S1. In addition to beef samples, the practical applicability of the as-prepared aptasensor was further validated by the detection of *P. aeruginosa* in tap water and raw milk. Under optimized experimental conditions, the standard addition method was employed by spiking the real samples with *P. aeruginosa* at concentrations of 10^3^, 10^5^, and 10^7^ CFU mL^−1^ by employing EIS measurements. The corresponding EIS responses were analyzed, and the obtained ΔRct values were used to plot the histograms (Figure S3). The results confirmed that the designed aptasensor demonstrated the robustness and reliability of the proposed sensing strategy in both tap water and raw milk. These findings underscore the biosensor’s applicability for the precise detection of *P. aeruginosa* in complex environments.

## 4. Conclusions

In summary, we reported the successful fabrication of a quaternary metal oxide nanocomposite FeCoCuNiO via the sol–gel combustion approach and subsequently doped with graphitic carbon nitride (g-C_3_N_4_). The structural and morphological study of synthesized materials was performed using XRD, FTIR, and SEM analysis, confirming the successful formation and doping of the fabricated materials. To further perform the electrochemical application, the synthesized material was then functionalized with a specific aptamer to design the aptasensor (apt/FeCoCuNiO-g-C_3_N_4_/GPE) for selective detection of *P. aeruginosa*. The EIS measurements demonstrated that the aptasensor exhibits sensitive detection of *P. aeruginosa* over a range of 1 × 10^1^–1 × 10^7^ CFU mL^−1^, with a limit of detection of 3.03 CFU mL^−1^. Furthermore, the stability tests confirm the long-term stability and reliable reproducibility of the designed aptasensor. The enhanced electrochemical performance is attributed to the synergistic interaction between g-C_3_N_4_ and FeCoCuNiO. Importantly, the achieved detection limit and wide dynamic range are in good competition with recently reported aptasensors for *P. aeruginosa* detection, thereby highlighting the significant potential of the designed aptasensor for the practical applicability of *P. aeruginosa* detection in contaminated food for food safety measurements.

## Data Availability

The original contributions presented in this study are included in the article/supplementary material. Further inquiries can be directed to the corresponding author.

## Supplementary Materials

The following supporting information can be downloaded at https://www.mdpi.com/article/10.3390/bios15100634/s1. S1. Instrument details; S2. Real sample preparation; S3. Calculation of the Limit of Detection (LOD); Figure S1. Optimization of aptamer concentration; Figure S2. This figure demonstrates Randles fitting of EIS data for different concentrations of bacteria; Figure S3. This figure represents the electrochemical monitoring of P. aeruginosa in three different analytes including tap water, raw milk and beef sample; Table S1. Detection of *P. aeruginosa* in a beef sample following the recovery method; Table S2. This table shows the detection of *P. aeruginosa* in the beef sample following the recovery method. References [43,44,45,46,47,48,49,50,51,52,53] are cited in the Supplementary Materials.
