# Supplementary material for: Real-Time and Selective Detection of Pseudomonas aeruginosa in Beef Samples Using a g-C3N4-Doped Multimetallic Perovskite-Based Electrochemical Aptasensor
Source: Biosensors (Basel). 2025 Sep 23;15(10):634. doi: 10.3390/bios15100634 (PMC12564328; doi:10.3390/bios15100634)
Supplement: Supplementary file 1 [file biosensors-15-00634-s001.zip › biosensors-3825571-supplementary.pdf]

biosensors

MDPI

Supplementary Materials

Real-Time and Selective Detection of *Pseudomonas Aeruginosa* in Beef Samples Using a g-C_3N_4-Doped Multimetallic Perovskite-Based Electrochemical Aptasensor

Sarah S. Albalawi [1], Naeem Akhtar [2] and Waleed A. El-Said [1,*]

[1] Department of Chemistry, College of Science, University of Jeddah, P.O. 80327, Jeddah 21589, Saudi Arabia
[2] Institute of Chemical Sciences, Bahauddin Zakariya University (BZU), Multan 60800, Pakistan
* Correspondence: waahmed@uj.edu.sa

S1. Instrument details

The instruments used during this work were Fourier Transform Infrared Spectroscopy (FT-IR Spectrometer, Burker), X-ray diffraction (XRD) Rigaku, Mini flex-II-Japan by means of Cu Kα (with a scan angle of 5^0-80^0, at 40 kV, 40 mA, and (2q)). Scanning Electron Microscope SEM was carried out by using Apreo S from ThermoFischer Scientific Eindhoven. Additionally, electrochemical application was performed using Potentiostat, (Gamry Reference 1010E), consisting of three reference electrodes (Ag/AgCl), as well as working (graphitic pencil electrode) and counter electrodes (platinum wire).

S2. Real Sample Preparation

The beef sample was prepared by following the strategy already reported [1]. Initially, a fresh beef sample was obtained from a local market in Multan for P. aeruginosa analysis. Initially, 5 g of the beef sample was weighed and transferred into a clean extraction vessel. The sample was treated with 40 mL of ethyl acetate in separate aliquots. The mixture was shaken vigorously for 20 min, followed by centrifugation at 10,000 rpm for 15 min. The supernatant was collected in a 50 mL container. The remaining solid residue was subjected to a second extraction using an additional 40 mL of ethyl acetate under the same conditions. The supernatants from both extraction steps were then combined. To adjust the pH for optimal detection, 1 M NaOH solution was added to the combined extract until a pH of 6–7 was achieved. The resulting solution was then subjected to EIS under optimized electrochemical conditions for the quantitative determination of P. aeruginosa in the beef sample.

S3. Calculation of Limit of Detection (LOD)

The limit of detection (LOD) was calculated using Equation S1, in accordance with the previously reported literature:

$$LOD = F \times SD/b \quad (S1)$$

where F is a factor of 3.3, SD represents the standard deviation of the ordinate intercept, and b is the slope of the regression line. Further, the sample size (n) used to calculate SD is 7.

 https://doi.org/10.3390/bios15100634

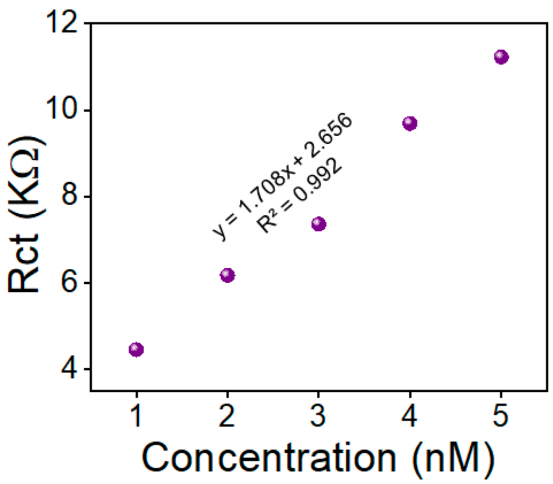

Figure S1. Optimization of aptamer concentration.

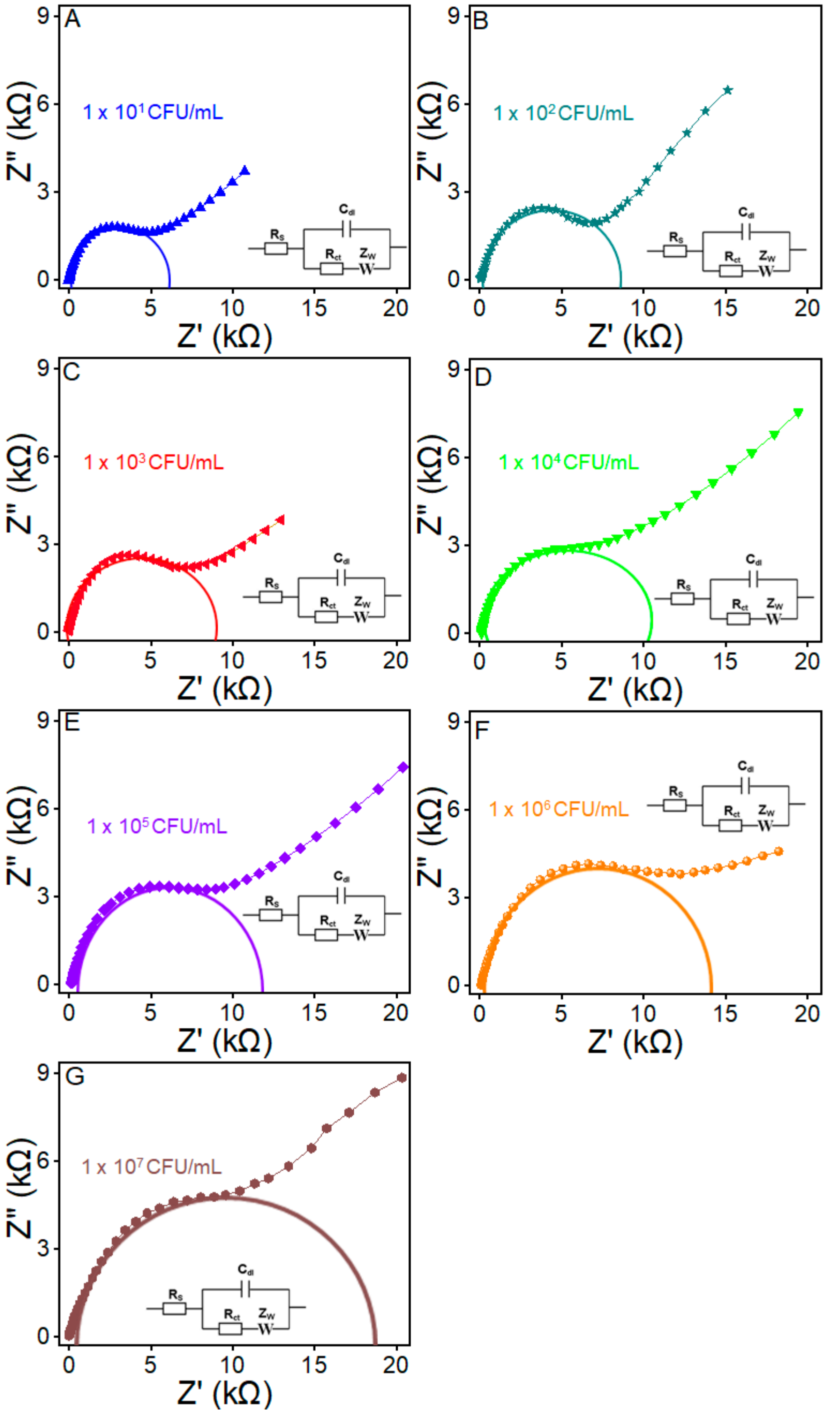

Figure S2. This figure demonstrates Randles fitting of EIS data for different concentrations of bacteria.

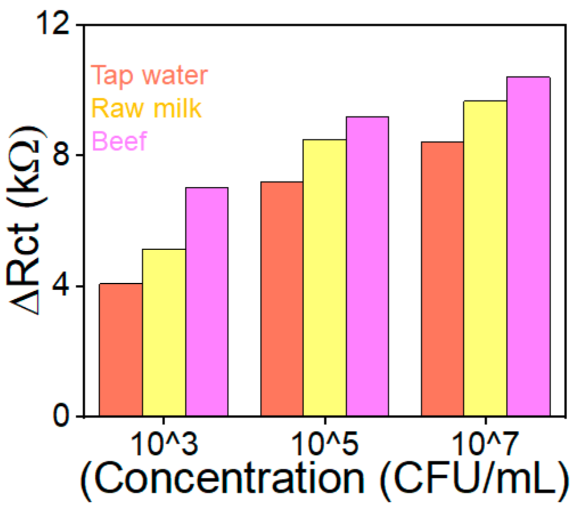

Figure S3. This figure represents the electrochemical monitoring of P. aeruginosa in three different analytes including tap water, raw milk and beef sample.

Table S1. This table indicates the superior detection limit of synthesized material for *P. aeruginosa.*

Materials	Strategy	Matrix	Aptamer Concentration	Linear Range	LOD (CFU mL^{-1})	Reference
Aptamer/AuNP-modified QCM chip	Label-free QCM-TSM	Milk, carrot Juice	-	-	68 (milk), 46 (carrot juice)	[2]
PDA@Co–NC-NPs/GPE aptasensor	Electrochemical	Spiked cow's milk	0.1–0.3 µM	10^1-10^6	2.8	[3]
AgNPs–rGO nanocomposite	Electrochemical	Water, milk	-	10^1-10^6	3	[4]
N-doped MWCNTs/AgNPs composite	Electrochemical	Buffer	-	10^{-1}-10^6	0.0798	[5]
Nanoporous gold electrodes	Electrochemical	Human serum	0–1 µM	2.5 to 140 pM	0.25 pM	[6]
F23 aptamer + hairpin DNA probe	Fluorescence assay with SDA	Culture media	-	-	1.6	[7]
GO sheets	Fluorescence resonance	Pork samples	60–140 nM	100–700 nM	9.6 nM	[8]
Recombinant LecB on NPG/GCE	Electrochemical	Milk, human serum	-	10-10^6	10	[9]
TWJ aptamer probe	fluorescent biosensor with TWJ-mediated SDA	Clinical samples	-	10-10^5	4.12	[10]
Apt13 ssDNA aptamer (SELEX-derived)	fluorescence quenching	Water samples	-	10^1-10^8	2.0	[11]
FeCoCuNiO–g-C_3N_4/Apt on GPE	Electrochemical	Beef (spiked)	1-5 nM	10^1-10^7	3.3	This work

Table S2. This table shows the detection of *P. aeruginosa* in the beef sample following the recovery method.

Sr. No	Added (CFU/mL)	Found (CFU/mL)	R.S.D (%)	R.E (%)	R (%)
1	10^3	0.98×10^3	1.42	2	98
2	10^5	1.02×10^5	1.40	2	102
3	10^7	1.04×10^7	2.77	4	104

References

1. Mohammad-Razdari, A.; Ghasemi-Varnamkhasti, M.; Izadi, Z.; Rostami, S.; Ensafi, A.A.; Siadat, M.; Losson, E. Detection of sulfadimethoxine in meat samples using a novel electrochemical biosensor as a rapid analysis method. *J. Food Compos. Anal.* **2019**, *82*, 103252.
2. Spagnolo, S.; Davoudian, K.; Franier, B.D.; Kocsis, R.; Hianik, T.; Thompson, M. Nanoparticle-Enhanced Acoustic Wave Biosensor Detection of Pseudomonas aeruginosa in Food. *Biosensors* **2025**, *15*, 146. doi:10.3390/bios15030146.
3. Yaqub, B.; Sarfraz, S.; Zulfiqar, A.; Wara, T.U.; Rasheed, S.; Ansari, S.H.; Hanif, S.; Khan, S.U.; Shah, M.; Akhtar, N. Development of polydopamine functionalized Co-EDTA complex-based electrochemical aptasensor for precise monitoring of Pseudomonas aeruginosa. *New J. Chem.* **2025**, *49*, 11107-11114, doi:10.1039/D5NJ00760G.
4. Wei, L.; Luo, S.; Zhou, W.; Ren, B.; Li, M.; Liang, L.; Li, X.; Wei, G. Rapid detection of Pseudomonas aeruginosa by glycerol one-pot RAA/CRISPR-Cas12a method. *Front. Chem.* **2025**, *13*,1654270. doi:10.3389/fchem.2025.1654270.
5. Li, J.; Sun, A.; Lai, H.; Li, C.; Li, H.; Yang, Z.; Pan, P.; He, J.; Zhang, R.; Wang, C. Ultrasensitive electrochemical aptasensor for Pseudomonas aeruginosa detection using N-doped MWCNTs/AgNPs nanocomposite. *Bioelectrochemistry* **2025**, *166*, 109031, doi:https://doi.org/10.1016/j.bioelechem.2025.109031.
6. Pourbahram, B.; Mansouri Majd, S.; Shamsipur, M. DPV and EIS-based ultrasensitive aptasensor for VEGF165 detection based on nanoporous gold platform modified with SH-aptamer. *Sens. Bio-Sens. Res.* **2025**, *47*, 100766, doi:https://doi.org/10.1016/j.sbsr.2025.100766.
7. Liu, L.; Li, J.; Wang, Z.; Yao, Z.; Cao, J. Sensitive Pseudomonas aeruginosa analysis based on aptamer-based target recognition mediated dual self-hybridization. *Microchem. J.* **2025**, *213*, 113687, doi:https://doi.org/10.1016/j.microc.2025.113687.
8. Xiao, S.; Sun, L.; Kang, M.; Dong, Z. A label-free aptasensor for clenbuterol detection based on fluorescence resonance energy transfer between graphene oxide and rhodamine B. *RSC Adv.* **2022**, *12*, 32737-32743, doi:10.1039/D2RA06260G.
9. Liu, R.; Cai, T.; Huang, Z.; Zhu, Q.; Wang, X. Novel ultrasensitive impedimetric biosensor for rapid detection of Pseudomonas aeruginosa via recombinant lectin-functionalized nanoporous gold biointerface. *Biosens. Bioelectron.* **2025**, *288*, 117788, doi:https://doi.org/10.1016/j.bios.2025.117788.
10. Wang, X.; Hu, J. Target recognition initiated reverse hybridization mediated cascade amplification for sensitive Pseudomonas aeruginosa analysis. *Biotechnol. Lett.* **2025**, *47*, 69, doi:10.1007/s10529-025-03612-5.
11. Li, X.; Cheng, Y.; Yang, K.; Liu, B.; Li, C. Selection and Application of Specific Nucleic Acid Aptamers for the Detection of Pseudomonas aeruginosa. *Food Sci.* **2025**, *46*, 322-328, doi:10.7506/spkx1002-6630-20240921-167.
